# 1-Isobutanoil-2-isopropylisothiourea Phosphate, T1082: A Safe and Effective Prevention of Radiotherapy Complications in Oncology

**DOI:** 10.3390/ijms23052697

**Published:** 2022-02-28

**Authors:** Marina Filimonova, Alina Saburova, Ljudmila Shevchenko, Victoria Makarchuk, Anna Shitova, Olga Soldatova, Vitaly Rybachuk, Alexander Kosachenko, Kirill Nikolaev, Grigory Demyashkin, Vyacheslav Saburov, Sergey Koryakin, Petr Shegay, Andrey Kaprin, Sergey Ivanov, Alexander Filimonov

**Affiliations:** 1 A. Tsyb Medical Radiological Research Center—Branch of the National Medical Research Radiological Center of the Ministry of Health of the Russian Federation, 249036 Obninsk, Russia; alinasamsonova.515@gmail.com (A.S.); schew.ludmila@yandex.ru (L.S.); vikymakarchuk@mail.ru (V.M.); annaredrose@mail.ru (A.S.); 89208861291@mail.ru (O.S.); rybachukvitaliy@gmail.com (V.R.); br.shepard@list.ru (A.K.); nireallki@gmail.com (K.N.); dr.dga@mail.ru (G.D.); vosaburov@gmail.com (V.S.); korsernic@mail.ru (S.K.); oncourolog@gmail.com (S.I.); filimonov_alex@mail.ru (A.F.); 2 National Medical Research Radiological Center of the Ministry of Health of the Russian Federation, 249036 Obninsk, Russia; dr.shegai@mail.ru (P.S.); kaprin@mail.ru (A.K.); 3 Medical Institute (RUDN University), Peoples’ Friendship University of Russia, 117198 Moscow, Russia

**Keywords:** radiation-induced tissue toxicity, radiotherapy, selective radiation protection, pharmacological safety

## Abstract

The radioprotective effects of a new 1-isobutanoil-2-isopropylisothiourea derivative named T1082 are presented. Research methods included toxic characteristics, radioprotective activity (Till–McCulloch’s test and 30-day survival test) in γ-ray total-body-irradiated mice, and a clinical and histological study of the effect of T1082 on acute radiation skin reactions (RSR) in rats after a single or fractionated β-ray local irradiation. T1082 is more effective than its analogue, the NOS inhibitor T1023, at low concentrations and doses (1/12–1/8 LD_10_), both parenterally and intragastrically. In this case, its therapeutic index (LD_50_/ED_50_) reaches 30, and the optimal radioprotective doses (ED_84–98_—141–224 mg/kg) are an order less than the maximum tolerated doses—1/16–1/10 LD_10_. These properties allowed T1082, at a low intragastrical dose (160 mg/kg; 1/14 LD10), to significantly limit the severity of acute RSR after single (40 Gy) and fractionated (78 Gy) β-ray irradiation. The results confirm T1082 as one of the safest emergency radioprotectors and indicate the prospects for its further development as a pharmacological agent for the prevention of RT complications.

## 1. Introduction

Radiation therapy (RT) remains one of the main treatments for many common types of cancer. Currently, about 50–60% of all cancer patients receive various types of RT during treatment [1,2]. However, despite continuous progress over the past 100 years in the field of oncology and radiology, the use of RT is still associated with significant risks of toxic effects. Acute or long-term complications due to radiation damage to normal tissues develop in 10–15% of RT cases (and in some tumor locations—in 40%) [3,4,5]. Such complications not only hinder the treatment of oncological diseases, but, in severe cases, are able to independently determine a negative prognosis, as well as lead to the development of internal organ failure and death [6,7,8]. It is obvious that, in order to minimize such threatening risks, it is necessary to both further improve medical radiological sources and methods of personal RT planning, and to develop new approaches to the pharmacological prevention and treatment of radiation pathologies.

The complex pathogenesis of acute and, especially, late radiation injuries, occurring with the participation of chronic immune and inflammatory reactions and fibrogenesis processes, provides a significant number of targets for therapeutic interventions [3,6,9,10]. At the same time, in general, regardless of organ-specific features, the single pathogenetic basis for such pathologies is primarily radiation-induced damage to molecular structures. In this regard, a fairly reasonable approach to the pharmacological prevention of RT complications can be the use of ‘direct’ radioprotectors that are able to limit the primary alteration of molecular targets by free-radical radiolysis products at the physical and physico-chemical stages of radiation injury.

We have previously shown that some linear and cyclic N,S-substituted isothioureas are effective competitive inhibitors of nitric oxide synthases (NOS) and have a pronounced vasoconstrictive, vasopressor effect [11,12,13]. The vasoconstrictive effect of these NOS inhibitors at relatively high doses is accompanied by reflex changes in hemodynamics (decrease in cardiac output) that can induce transient tissue hypoxia and provide a radioprotective effect [14,15,16].

**Figure 1:**
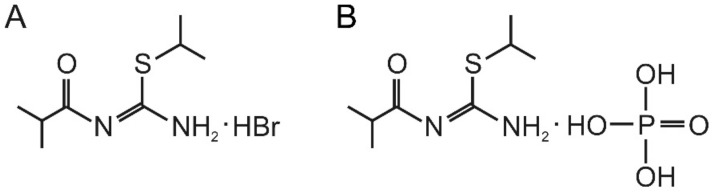
Structural formulas of the studied compounds. (**A**) 1-isobutanoyl-2-isopropylisothiourea hydrobromide (compound T1023); (**B**) 1-isobutanoyl-2-isopropylisothiourea phosphate (compound T1082).

Among the studied derivatives, the compound T1023, 1-isobutanoyl-2-isopropylisothiourea hydrobromide, had a high radioprotective activity (Figure 1A). T1023, when administered preventively at safe doses (1/5–1/4 LD_10_; 60–75 mg/kg), provides effective prophylaxis (dose modifying factor (DMF)—1.6–1.9) of H-ARS and G-ARS in mice [17,18]. The T1023 compound is also effective in the prevention of RT complications. In radioprotective doses, it significantly limits (DMF—1.4–1.7) the frequency and severity of acute (radiation skin burns in mice and rats) and late (radiation pneumofibrosis in rats) radiation injuries. Moreover, in RT models of transplanted tumors of various histogenesis (solid Ehrlich carcinoma in mice and M-1 sarcoma in rats), T1023 fully and selectively protects normal tissues, without weakening the antitumor effects and the overall effectiveness of RT neoplasia [19,20,21].

At the same time, these studies also revealed the features of T1023 radioprotective activity, which can negatively affect its effectiveness and safety. Namely, a narrow range of its effective radioprotective doses—1/5–1/3 LD_10_ (60–90 mg/kg)—is close enough to its toxic threshold. Already at doses of 1/8 LD_10_ (40 mg/kg), the effect of T1023 is usually weak [17,18]. Such features, in our opinion, retain the risks of both the development of negative effects and T1023 ineffectiveness, due to the influence, for example, of metabolic disorders or pathologies that can significantly distort the pharmacokinetics of the active substance [22]. In this regard, before starting the practical development of T1023, we considered it expedient to search for a more acceptable ‘transport’ form of the active substance among its closest analogues—in the series of 1-isobutanoyl-2-isopropylisothiourea salts with various inorganic and organic acids [23].

The results of these studies showed that changing the salt-forming acid from hydrobromide to hydroiodide, hydrochloride, oxyethylidenediphosphonate, phosphonate, maleate, and malonate had a negative effect on the radioprotective activity of 1-isobutanoyl-2-isoprolysothiourea salts. In a wide dose range (25–130 mg/kg), the radioprotective effect of such compounds was, to varying degrees, lower (by 10–40%) than the effects of the hydrobromide salt (T1023) at equimolar doses. At the same time, the radioprotective activity of 1-isobutanoyl-2-isoprolysothiourea phosphate (compound T1082; Figure 1B) was qualitatively different. Unlike other compounds, this salt, at all doses, realized a higher (by 15–60%) radioprotective activity than T1023. Moreover, the most pronounced radioprotective advantage of compound T1082 was observed at low doses (1/12–1/8 LD_10_; 25–40 mg/kg), in which T1023 is almost or completely ineffective. These data suggest T1082 has a wider range of effective doses, which makes it possible to correct the shortcomings of T1023 and significantly increase the efficacy and safety of the developed means.

In this regard, we conducted a detailed study of T1082 toxic properties, radioprotective activity, and its ability to prevent acute radiation damage. The results, in general, allow a fairly objective assessment of its prospects for further development as a means of preventing RT complications.

## 2. Results

### 2.1. Changing the Salt-Forming Acid from HBr to H_3_PO_4_ Does Not Change the Toxic Characteristics of the Active Substance

In toxicological studies, the compound T1082, with a single intraperitoneal (i.p.) administration at doses of 300–470 mg/kg, caused intoxication in mice, the dynamics and manifestations of which reproduced the acute toxic effects of the compound T1023 at the same doses and method of application [18]. Adynamia rapidly developed. At doses of 350 mg/kg and above, respiratory arrhythmia, tremor, and clonic convulsions increased, at the peak of which respiratory arrest and death occurred. The lethal effect of T1082 developed in the first 20–60 min after administration. The manifestations of intoxication in surviving mice began to subside after 1.5–2 h and completely regressed 3–4 h after administration. A day later, and in the next 15 days of observation, the surviving animals did not differ from intact, healthy mice in appearance, behavior, motor activity, reactions to stimuli, and body weight dynamics. Macroscopic changes in the internal organs of dead and surviving mice were not observed.

According to the results of toxicometry, the estimates of the maximum tolerated dose (LD_10_) and the average lethal dose (LD_50_) of the T1082 compound (i.p. administration) to mice were 321 and 403 mg/kg, respectively. A comparison of T1082 and T1023 toxicity parameters, with i.p. administration, did not reveal significant differences—for all indicators, the differences did not exceed the statistical errors of estimates and were within 5–8% (Table 1). With intragastric (i.g.) administration of T1082, the sensitivity of mice to its toxic effect decreased by 6.5–7 times—the estimates of LD_10_ and LD_50_ were 2290 and 2638 mg/kg, respectively. In this case, intoxication in mice had the same character, but developed less rapidly, and the lethal effect of T1082 was more delayed to 2–4 h after administration.

Thus, the toxicological data indicated that changing the salt-forming acid from hydrobromide to phosphate had no significant effect on the toxic characteristics of 1-isobutanoyl-2-isopropylisothiourea salts. Both compounds belong to the “moderately hazardous” class [24] with very similar quantitative characteristics.

### 2.2. Changing the Salt-Forming Acid from HBr to H_3_PO_4_ Allows the Active Substance to Realize a Significant Effect at Low Doses and Concentrations

At the same time, such a molecular structural change in the active substance had a significant effect on the radioprotective activity of the salts of this isothiourea.

In the first series of the radiobiological study, two independent experiments were made to compare T1023 and T1082 administered i.p. 30 min before irradiation in equimolar doses that corresponded to the levels of 1/18, 1/12, 1/8, 1/4, and 1/3 LD_10_ on the number of endogenous splenic colony forming units (CFU) in F_1_ (CBA×C57BL6j) mice on the 8^th^ day after γ-ray total body irradiation (TBI) at a dose of 5 Gy.

**Figure 2:**
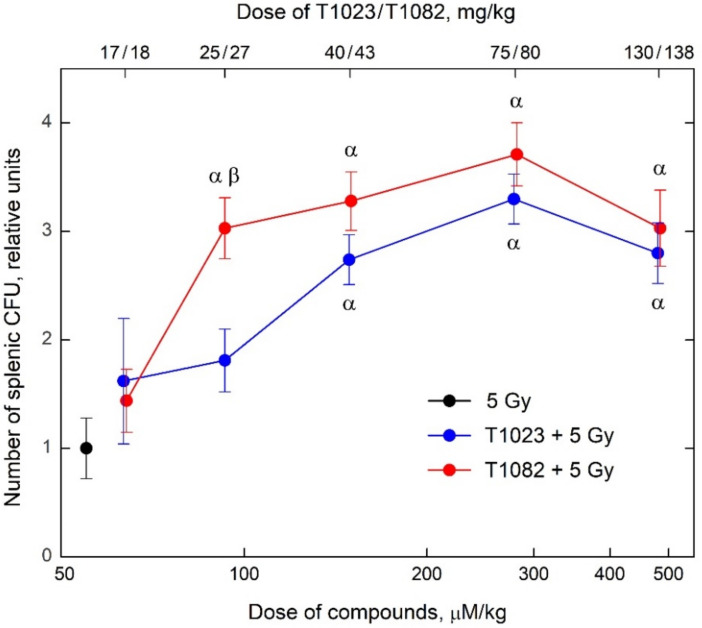
The effect of compounds T1023 and T1082 (single, i.p., 30 min before irradiation) in equimolar doses corresponding to 1/18, 1/12, 1/8, 1/4, and 1/2 LD_10_ on the number of endogenous splenic CFU in F_1_ (CBA×C_57_BL_6j_) mice on day 8 after 5 Gy γ-ray TBI. Data represent the combined results of two independent experiments—data for each experiment are normalized to the mean number of CFU in the irradiated control (see Section 4.5) and graphical deviations correspond to SD (*n* = 32–34 per point). α, significantly different vs. irradiated control mice (for T1023: *p* = 0.00734, *p* = 0.00012, and *p* = 0.00487; for T1082: *p* = 0.00263, *p* = 0.00028, *p* = 0.00004, and *p* = 0.00180, respectively). β, significantly different between mice treated with T1023 and T1082 at equimolar doses (*p* = 0.00386).

In these experiments, T1023 stably realized its characteristic dose dependence of radioprotective activity [17,18] (Figure 2). There was no effect in the region of 1/18–1/12 LD_10_ (17–25 mg/kg), while a significant effect appeared at 1/8 LD_10_ (40 mg/kg) and reached a maximum in the region of 1/4 LD_10_ (75 mg/kg). At the same time, in both experiments, the dose dependence of T1082 radioprotective activity was significantly different. Its radioprotective activity was absent only in the region of 1/18 LD_10_ (18 mg/kg), and in the range of 1/12–1/4 LD_10_ (27–80 mg/kg), the effect of T1082 was highly significant (*p* = 0.49–0.85). The difference in T1082 radioprotective activity in these experiments was also confirmed by a statistical comparison of the effects of T1023 and T1082 in equimolar doses. If in the area of 1/8 LD_10_ the difference remained at the level of a trend (*p* = 0.05255), then in the area of 1/12 LD_10_, it was characterized by a high level of significance (*p* = 0.00386).

The radioprotective ability of T1082, when used preventively at low doses (1/12–1/8 LD_10_), was even more contrasting in a comparative study of the effects of T1023 and T1082 (i.p., 30 min before TBI), at equimolar doses corresponding to levels of 1/12, 1/8, and 1/4 LD_10_, on the 30-day survival of F_1_ (CBA×C57BL6j) mice after γ-ray TBI at a dose of 9.5 Gy.

**Figure 3:**
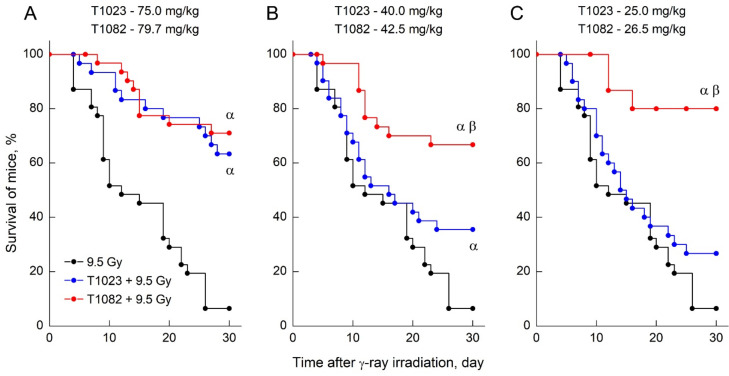
The effect of compounds T1023 and T1082 (single, i.p., 30 min before irradiation) in equimolar doses corresponding to 1/4 LD_10_ (**A**), 1/8 LD_10_ (**B**), and 1/12 LD_10_ (**C**) on the survival of F_1_ (CBA×C_57_BL_6j_) mice after 9.5 Gy γ-ray TBI. Data represent the combined results of two independent experiments (*n* = 15–31 per group). Survival was plotted using the Kaplan–Meier method. α, significantly different vs. irradiated control mice (for T1023: *p* = 0.00008 and *p* = 0.03158; for T1082: *p* = 0.00006, *p* = 0.00022, and *p* = 0.00003, respectively). β, significantly different vs. T1023-treated mice (*p* = 0.00808 and *p* = 0.00049, respectively).

In these experiments, T1023 also realized the dose dependence of radioprotective activity expected for this test [17,18] (Figure 3). At an optimal radioprotective dose of 1/4 LD_10_ (75 mg/kg), it was highly effective (30-day survival—63%), at a dose level of 1/8 LD_10_ (40 mg/kg), there was a minimal significant effect (30-day survival—36%), and at a dose level of 1/12 LD_10_ (25 mg/kg), no significant effect was observed (30-day survival—27%). At the same time, T1082 provided pronounced radiation protection (30-day survival—67–80%), equal in effectiveness (*p* = 0.53–0.76) in the entire dose range 1/12–1/4 LD_10_ (27–80 mg/kg). Moreover, the effects of T1082 at doses of 1/12 and 1/8 LD_10_, at a high level of significance (*p* = 0.00049, *p* = 0.00808), exceeded the effects of T1023 at the corresponding doses.

Thus, the results of two independent experiments on various tests confirmed the data of preliminary screening with high statistical significance. They indicate that changing the salt-forming acid from HBr to H_3_PO_4_ positively modifies the radioprotective activity of 1-isobutanoyl-2-isopropylisothiourea salts, allowing the active substance, in low doses (concentrations), to realize a significant effect. From the standpoint of pharmacology, the consequences of such a modification are significant. A two-fold increase in the range of effective radioprotective doses (radioprotective latitude) is registered—from 60–90 mg/kg for the hydrobromide salt to 27–90 mg/kg for phosphate salt. It is important that the increase in this range is realized due to the expansion into the region of lower, safer doses—1/12–1/8 LD_10_. In this case, a 2–3-fold increase in the distance from the toxic threshold when using T1082 reduces the risks of developing negative effects without a loss of radioprotective efficiency, and an increase in radioprotective latitude potentially reduces the risks of low-efficiency.

### 2.3. T1082: Perhaps the Safest Emergency Preventive Radioprotector

**Figure 4:**
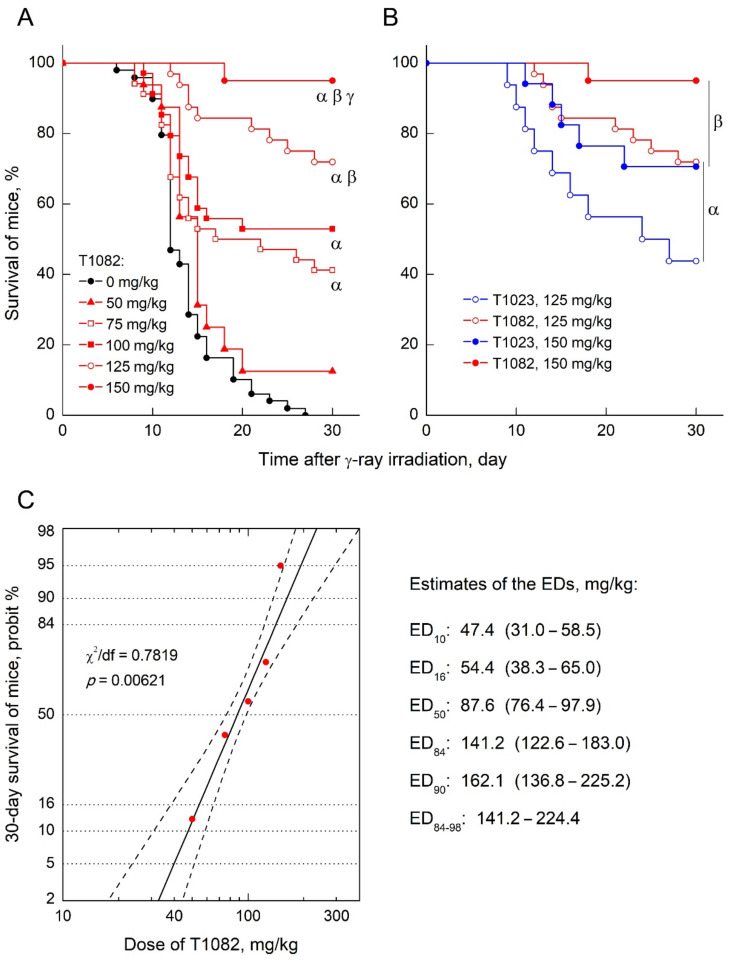
The effect of compounds T1023 and T1082 (single, i.g., 30 min before irradiation) on the survival of F_1_ (CBA×C_57_BL_6j_) mice after 9 Gy γ-ray TBI. (**A**) Effects of compound T1082 at doses of 50–150 mg/kg. Data represent the combined results of two independent experiments (*n* = 20–34 per group). Survival was plotted using the Kaplan–Meier method. α, significantly different vs. irradiated control mice (*p* = 0.01222, *p* = 0.00943, *p* = 0.00007, and *p* < 0.00001, respectively). β, significantly different from the group treated at a dose of 75 mg/kg (*p* = 0.01370 and *p* = 0.00031). γ, significantly different from the group treated at a dose of 125 mg/kg (*p* = 0.00542). (**B**) Effects of compounds T1023 and T1082 at doses 125 and 150 mg/kg. Data represent the combined results of two independent experiments (*n* = 15–34 per group). Survival was plotted using the Kaplan–Meier method. α, significant difference in effects at a dose 125 mg/kg (*p* = 0.01683). β, significant difference in effects at a dose 150 mg/kg (*p* = 0.02685). (**C**) Dose dependence of the radioprotective efficacy of compound T1082 on the 30-day survival of mice (probit analysis). Graphic points represent experimental indicators of 30-day survival of mice (from (A)). Solid line, mean expected effective doses of compound T1082 (probit-logarithmic regression; *p* shows the significance of regression). Dashed lines, 95% confidence interval of expected effective doses. On the right are estimates of ED values with different radioprotective efficiencies (in brackets—95% confidence interval for estimates).

At the same time, the radioprotective effectiveness at low doses (concentrations) is able to provide T1082 advantages not only in safety, but also in applicability. In particular, it may increase the acceptability of such an active substance for i.g. administration, since in this case the processes of absorption from the gastrointestinal tract can prevent the formation of effective tissue concentrations [22]. In this regard, a comparative study of T1023 and T1082 effects with i.g. administration (i.g., 30 min before TBI) on 30-day survival of F_1_ (CBA×C_57_BL_6j_) mice after γ-ray TBI at a dose of 9 Gy was performed. It was found that T1082, when administered once at doses of 50–150 mg/kg i.g., exhibits a distinct dose-dependent radioprotective activity (Figure 4A). Its radioprotective effect increased from an insignificant level (30-day survival—17%), at a dose of 50 mg/kg, to an absolutely effective level (30-day survival—95%) at a dose of 150 mg/kg. Compound T1023, with the same route of administration at doses of 125 and 150 mg/kg, also had a radioprotective effect in these experiments (Figure 4B). However, at both doses, the effect of T1082 was significantly superior (*p* = 0.01683, *p* = 0.02685) to that of T1023. Further, the equal effect of T1082 upon i.g. administration was realized at significantly (20–40%) lower doses than T1023; the effect of T1023 at a dose of 125 mg/kg was equal in efficiency (*p* = 0.61–0.79) to that T1082 at doses of 75–100 mg/kg and the effect of T1023 at a dose of 150 mg/kg was equal in efficiency (*p* = 0.84) to that of T1082 at a dose of 125 mg/kg.

In addition, these data also allowed for the reliable statistical (*p* = 0.00621) quantitative characterization of the dose dependence of T1082 radioprotective efficacy when administered i.g. (Figure 4C). According to the results of the probit analysis, the minimum radioprotective dose of T1082 (ED_16_) is 54.4 mg/kg, the average effective dose (ED_50_) is 87.6 mg/kg, and the range of optimal radioprotective doses (ED_84-98_—probit in the interval 6.0–7.0) is in the range of 141.2–224.1 mg/kg (for mice, i.g. administration). When analyzing the obtained estimates of EDs and comparing them with the data of toxicological studies (Table 1), attention was drawn to the difference in the effect of switching from parenteral to intragastric administration of T1082 on mice sensitivity to its toxic and radioprotective effects. Thus, if mice sensitivity to the T1082 radioprotective effect decreased by 2.5–5 times after i.g. administration in comparison with i.p. (ED_84-98_ increased from 27–90 mg/kg to 141–224 mg/kg), then the sensitivity to the T1082 toxic effect decreased significantly more—by 6.5–7 times (LD_10_ and LD_50_ increased from 321 and 403 mg/kg to 2290 and 2638 mg/kg, respectively). That is, the transition to i.g. administration of T1082 increased the level of its safety, significantly (by at least 40%) expanding the distance between the optimal radioprotective doses and the toxic threshold.

These data suggest the i.g. route of T1082 administration as not only acceptable, but probably the most preferred. In particular, its calculated therapeutic index (LD_50_/ED_50_) is 30, and its optimal radioprotective doses (ED_84-98_) are more than an order of magnitude less than the maximum tolerated doses for i.g. administration—1/16–1/10 LD_10_ (Table 2). Such pharmacological ratios show the advantage of T1082 in comparison with many known radioprotective agents [25]. This allows for its classification as one of the safest radioprotectors with an emergency, preventive effect.

### 2.4. T1082: An Effective and Safe Means for the Prevention of Radiotherapy Complications

To assess the prospects of T1082 as a means of preventing complications of RT, clinical, morphological, and histological studies of its effect on the development of acute radiation skin reactions (RSR) were carried out in Wistar rats after β-ray (10 MeV) local irradiation (LI) of the lateral surface of the right thigh. The effects were studied under two variants of radiation exposure: single LI at dose of 40 Gy and fractionated LI at a total dose of 78 Gy (6 fractions at a dose of 13 Gy with 24 h intervals). Compound T1082 was administrated once or 6 times (i.g., 30 min before each LI) at a dose of 160 mg/kg, which in this case, is about 1/14 LD_10_ and corresponds to ED_90_. Biological material for morphological studies (in untreated rats—thigh tissues; in control and T1082-treated rats—thigh tissues, stomach, and small intestine) was isolated on the 15th day after the start of fractionated exposure and on the 11th day after a single exposure.

Single and six-fold injections of compound T1082 at 160 mg/kg, i.g., were tolerated easily and painlessly by rats. The only change that was noted in these animals was moderate physical inactivity during the first 60–90 min after injection. No other manifestations of intoxication, disturbances of sensitivity, behavior, food activity, and body weight dynamics were noticed.

**Figure 5:**
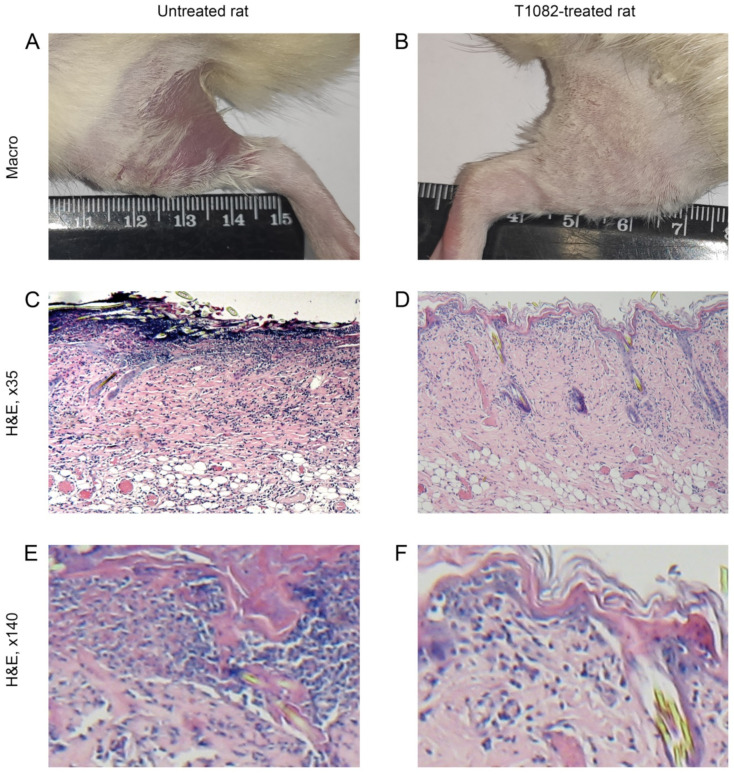
The effect of compound T1082, at a dose of 160 mg/kg i.g. (1/14 LD_10_), by single administration, on the morphology of rat hip tissues on day 11 after β-ray LI at a dose of 40 Gy. (**A**,**B**) Macroscopic pictures. (**A**) Untreated rat. The RSR corresponds to 3 degrees according to the RTOG scale: severe hyperemia and edema, extensive epilation, and confluent exudative epidermitis, spreading to the inguinal area. (**B**) T1082-treated rat. The RSR corresponds to 1 degree according to the RTOG scale: weak epilation, moderate erythema, single petechiae (less than 1 mm), and intensive dry desquamation. (**C**,**D**) General histological picture of thigh tissues (H&E; ×35). (**C**) Untreated rat. The epidermis is destroyed and, in some places, thin-walled scrabs form from necrotic masses infiltrated by neutrophils; the dermis is edematous, collagen fibers are swollen, and the hair follicles are destructured, but the hypodermis is little changed and the subcutaneous vessels have a preserved structure. (**D**) T1082-treated rat. The epidermis is preserved, but unevenly thickened due to the proliferation of basal and spiny cells; the stratum corneum is stratified in places and, in the moderately edematous dermis, intact appendages with a thickened outer epithelium are visible, while subcutaneous adipose tissue is not changed. (**E**,**F**) Fragments of C-D, respectively (H&E, ×140). (**E**) Untreated rat. Intensive leukocyte infiltration extending to the external root sheath, replacement of the hair follicle. (**F**) T1082-treated rat. Moderate infiltration of fibroblasts and lymphocytes in the upper dermis.

According to clinical observations, the initial development of RSR in untreated and T1082-treated rats after single β-ray LI at dose of 40 Gy did not differ significantly. By day 3–4 after LI, moderate erythema and dry desquamation developed in these rats. From day 6 after LI, RSR severity in untreated rats increased. On the background of increasing edema, the epidermitis acquired an exudative character. By the 11th day after LI in all untreated rats, RSR corresponded to grade 3 on the RTOG-95 scale [26]; confluent exudative epidermitis was observed, which was more spread over the inner surface of the irradiated thigh (Figure 5A). At the same time, there was no increase in the severity of the radiation burn in T1082-treated rats by this time. RSR remained within the 1st degree RTOG (significantly different with untreated rats; *p* = 0.02535); moderate hyperemia and intense dry epidermitis were observed, but without significant damage to the skin (Figure 5B).

A microscopic examination of the thigh tissues in untreated rats on day 11 after a single β-ray LI at dose of 40 Gy revealed the absence of the epidermis in the areas of the most pronounced lesion, as well as the formation of thin scabs consisting of necrotic masses infiltrated by neutrophils (Figure 5C,E). In the compacted dermis, remnants of the epithelium and skin appendages with replacement, moderate edema, and infiltration by lymphocytes and neutrophils were observed. The hypodermal layer is slightly modified. Most of the vessels in the subcutaneous tissue have a preserved structure.

A microscopic examination of the thigh tissues in T1082-treated rats on day 11 after a single β-ray LI at dose of 40 Gy revealed the almost complete preservation of the epidermis, which is unevenly thickened due to the growth of the basal layer and the layer of spiny cells (Figure 5D,F). In the moderately edematous dermis, preserved sebaceous glands and hair follicles with a thickened outer epithelial layer are visible, as is a moderate fibroblast infiltration of the upper dermis. The subcutaneous adipose tissue is not changed.

**Figure 6:**
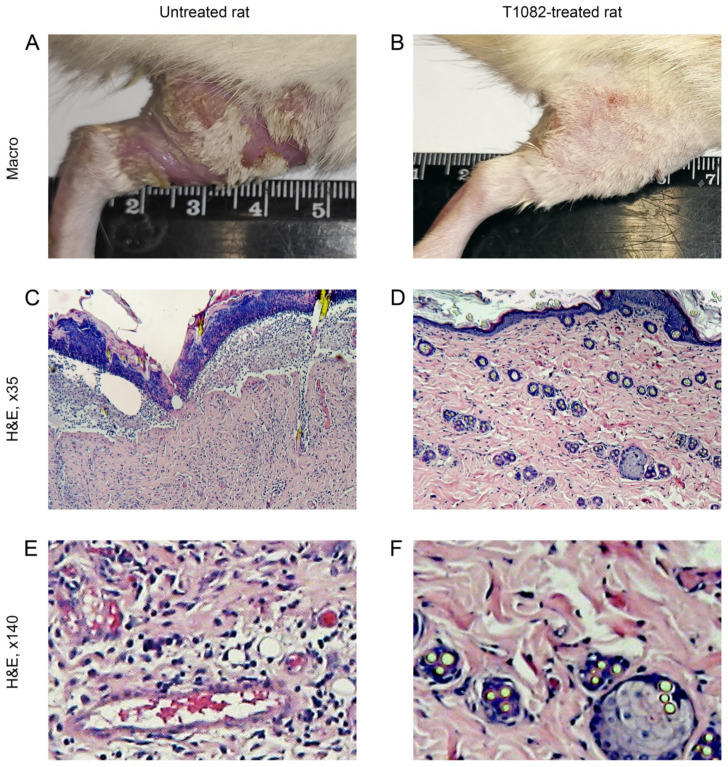
The effect of compound T1082 at a dose of 160 mg/kg (1/14 LD_10_), i.g., given by multiple administration, on the morphology of rat thigh tissues on the 15th day after the start of fractionated β-ray LI at a total dose of 78 Gy (6 fractions at a dose of 13 Gy with 24 h intervals). (**A**,**B**) Macroscopic pictures. (**A**) Untreated rat. The RSR corresponds to 4 degrees according to the RTOG scale: severe hyperemia and edema, confluent exudative epidermitis spread over the entire surface of the thigh, and a radiation ulcer (3 × 4 × 3 mm) on the knee joint with purulent contents. (**B**) T1082-treated rat. The RSR corresponds to 2 degrees according to the RTOG scale: epilation, hyperemia, and dry epidermitis, which, in some places, took on an islet wet form. (**C**,**D**) General histological picture of thigh tissues (H&E; ×35). (**C**) Untreated rat. The epidermis is missing and the papillary layer is intensively infiltrated with leukocytes and exfoliates, forming cavities filled with necrotic masses and leukocytes; destroyed follicles and sebaceous glands are observed in the reticular layer and the hypodermis is sharply expanded due to severe edema and loosening of the fibers. Vessels in adipose tissue are dilated and altered. Edema is present on the internal basement membrane, perimysium, and endomysium of the muscles. (**D**) T1082-treated rat. The epidermis is preserved, although it is unevenly thickened due to basal and spiny cell proliferation; in the moderately edematous dermis, intact appendages with a thickened outer epithelium are visible and, in the hypodermis, there is a slight perivascular edema, while muscle fibers are unchanged. (**E**,**F**) Fragments of C-D, respectively (H&E, ×140). (**E**) Untreated rat. Edema, loosening of collagen fibers, and leukocyte infiltration in the hypodermis; subcutaneous vessels are dilated, and, in some places, there is detachment of the endothelium and atresia of the lumen of the vessels by aggregated erythrocytes. (**F**) T1082-treated rat. Hypertrophy of the outer epithelial layer of hair follicles and sebaceous glands.

In untreated rats after fractionated LI at a total dose of 78 Gy, the development of RSR rapidly became severe. Already by the 7th day, on the background of severe edema, confluent exudative epidermitis developed in all these animals. Later, in some rats, it was complicated by local bleeding and ulceration. By the 15th day from the start of fractionated LI, RSR in untreated rats corresponded to grades 3–4 (Figure 6A)—hyperemia, severe edema, and widespread confluent exudative epidermitis were observed, which in 60% of such animals was complicated by superficial radiation ulcers with purulent discharge. An increase in RSR manifestations from the 7th day was also observed in T1082-treated rats. The edema increased and the epidermitis acquired an exudative character. By the 15th day from the start of fractionated LI, RSR in T1082-treated rats corresponded to grades 2–3 (significantly different with untreated rats; *p* = 0.03390) (Figure 6B)—hyperemia, moderate edema, and dry and insular moist epidermitis were observed, which in 30% of such animals acquired a confluent form on the inner surface of the thigh.

A microscopic examination of the thigh tissues in untreated rats on the 15th day from the start of fractionated LI showed no epidermal layer (Figure 6C). The papillary layer is smoothed and intensively infiltrated with polymorphonuclear leukocytes, exfoliated in places, forming cavities partially filled with necrotic masses, tissue fluid, and leukocytes. In the edematous dermis, loosened collagen fibers are observed and the destruction of hair follicles and sebaceous glands is noted. The hypodermal layer is significantly expanded due to edema and the loosening of collagen fibers. Vessels in adipose tissue are dilated, and endothelial exfoliation is observed in places, as is atresia of the lumen of small vessels with smoothed erythrocytes (Figure 6E). The internal basement membrane, perimysium, and endomysium of the muscles are edematous.

A microscopic examination of the thigh tissues in T1082-treated rats on day 15 from the start of fractionated LI show that the epidermal layer is almost completely preserved, although unevenly thickened due to the proliferation of basal and spiny cells (Figure 6D,F). Preserved sebaceous glands and hair follicles with a thickened epithelial layer are visible in the moderately edematous dermis. Moderate perivascular edema is noted in the hypodermis. The internal basement membrane and muscle fibers remain unchanged.

A comparison of the tissues of the irradiated thigh in untreated and treated rats indicated that the use of T1082 at a dose effective for ARS prevention, with single and fractionated LI, was accompanied by a significant decrease in the level of radiation injuries, as well as the faster regeneration and normalization of the histological structure of the affected skin and underlying tissues. Morphological data indicate that the effect of T1082 in this case limits damage and maintains the consistency and functional activity of cell populations critical for RSR development—cells of the basal layer, epithelium of the skin appendages, fibroblasts, and vascular endothelium [3,27].

**Figure 7:**
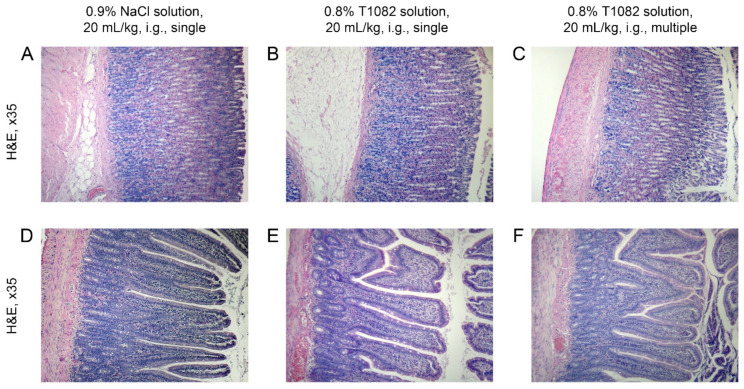
The effect of compound T1082, at a dose of 160 mg/kg, i.g., by single and multiple administration, on the histology picture of the tissues of the greater curvature of the stomach (**A**–**C**) and jejunum (**D**–**F**) on the 11th day after last administration (H&E; ×35). (**A**,**D**) A rat that received a single administration of 0.9% sodium chloride solution at a dose of 20 mL/kg. The histological structure of the tissues of stomach and jejunum corresponds to the age norm. (**B**–**E**) A rat that received a single administration of 0.8% T1082 solution at a dose of 20 mL/kg. The histological structure of all layers of the wall of the stomach and the wall of the jejunum corresponds to the norm. (**C**–F) A rat that received a multiple administration of 0.8% T1082 solution at a dose of 20 mL/kg. Pathological changes in the histostructure of the stomach and jejunum were not observed.

In favor of the promise of T1082, data on its safety is also testified. Compound T1082 did not appear to have a local damaging effect at the doses used, which effectively counteracted the development of RSR. Microscopic studies of stomach and jejunum tissues in irradiated T1082-treated rats after its single and multiple i.g. administration at a dose of 160 mg/kg did not reveal pathological changes in the histostructure of these organs (Figure 7).

## 3. Discussion

The scale and severity of the medical and socio-economic consequences of acute and late toxic effects of RT on tumors in recent decades have taken these problems beyond the scope of oncology and radiation medicine. Currently, specialists in various fields of biology and medicine pay considerable attention to the prevention and treatment of RT complications. The objects of such research are an extremely wide range of compounds with various pathophysiological and pharmacological activities, which are capable of limiting radiation alteration, modulating the processes of repair and cell death, the course of immune-inflammatory reactions and fibrogenesis, etc. [28,29,30].

As radioprotectors are able to prevent the formation of primary molecular damage by radiolysis products, its property to limit the frequency and severity of RT complications is quite expected. This ability has been experimentally shown for aminothiol and indolylalkyl radioprotectors [31,32], as well as antioxidants and free radical inhibitors [33,34,35]. However, despite this, to date, only one drug with such a mechanism of action (amifostine) is approved for use in humans as a means of preventing RT complications. This situation is primarily due to the low tolerance and potential toxicity for humans of many known radioprotectors. Thus, even amifostine has clinically significant limitations and requires the careful preparation and management of patients since it can cause significant negative effects that may require emergency correction [31,36].

In our research, we are trying to overcome the shortcomings of existing radioprotective agents and, it seems, have made some progress. Indeed, the originally developed agent based on the NOS inhibitor T1023 (1-isobutanoyl-2-isopropylisothiourea hydrobromide) demonstrates quite convincing results—at a relatively safe dose (75 mg/kg; i.p.; 1/4 LD_10_), it effectively counteracts (DMF—1.6–1.9) the development of H-ARS and G-ARS [17,18] and no less effectively (DMF—1.4–1.7) provides selective prevention of acute (RSR in mice and rats) and late (radiation pneumofibrosis in rats) RT complications without reducing antitumor effects [19,20,21].

In this study, we have shown that 1-isobutanoyl-2-isopropylisothiourea phosphate (compound T1082) is a more perfect (first of all, safer) analog. The results of independent experiments on various tests indicate that changing the salt-forming acid from HBr to H_3_PO_4_ positively modifies the radioprotective activity of 1-isobutanoyl-2-isopropylisothiorea salts, allowing the administration of the active substance in low doses (concentrations) to realize a significant effect. A two-fold increase in the range of effective radioprotective doses is observed—from 60–90 mg/kg for the hydrobromide salt to 27–90 mg/kg for the phosphate salt. An increase in this range is realized by expanding into the region of low, safer doses—1/12–1/8 LD_10_. In this case, the opportunity of using 2–3 times lower doses of T1082 without a loss of radioprotective efficacy significantly reduces the risks of possible negative effects.

Moreover, the safety level of T1082 increases significantly when administered intragastrically. In this case, the T1082 therapeutic index (LD_50_/ED_50_) reaches 30, and its optimal radioprotective doses (ED_84-98_—141–224 mg/kg) are an order of magnitude lower than the maximum tolerated doses—1/16–1/10 LD_10_. These data allow for the classification of T1082 as one of the safest radioprotectors with emergency, preventive action [25].

Both compounds, T1023 and T1082, have the same physiologically active substance—1-isobutanoyl-2-isopropylisothiourea. The results of morphological studies showed that compound T1082, preventively, i.g., 160 mg/kg (1/14 LD_10_), and compound T1023, preventively, i.p., 75 mg/kg (1/4 LD_10_) [21], had a similar effect on the development of acute radiation damage to the skin in rats after single local irradiation at dose of 40 Gy. The use of T1023 and T1082 at doses effective for ARS prevention was accompanied by a significant decrease in the level of radiation injuries, rapid epithelialization of the damage zone, and normalization of the histological structure. Additionally, in both cases, histology data indicated that the protective effect was realized by limiting the damage and maintaining the functional activity of critical cell populations [3,27]—cells of the epidermis basal layer, hair follicles epithelium, vascular endothelium, and fibroblasts. At the same time, T1082 provides such effects at doses that are much safer. The efficacy and safety of T1082 is also confirmed by research data with fractionated local irradiation at a dose of 78 Gy. Multiple i.g. administration of T1082 significantly counteracted the development of severe RSR in rats, without causing any negative effects.

## 4. Materials and Methods

### 4.1. Animals

Male outbred ICR mice (CD-1; 4–5 months old, 27–31 g body weight), male F_1_ (CBA × C57BL6j) mice (2–3 months old, 19–23 g body weight), and male Wistar rats (3–4 months old, 200–270 g body weight) were used in these studies. Animals were purchased from the Biomedical Technology Scientific Center of Federal Biomedical Agency of Russia (Moscow). The animals were housed in T-3 and T-4 cages under natural light conditions with forced ventilation 16 times/h, at a room temperature 18–20 °C and relative humidity 40–70%. The animals had free access to water and rodents PK-120-1 feed (Laboratorsnab Ltd., Moscow, Russia). Animal studies were approved by the A. Tsyb Medical Radiological Research Center (MRRC) Ethical Committee and were performed in accordance with generally accepted standards for the animal treatment, based on standard operating procedures of the A. Tsyb MRRC, in accordance with the rules and requirements of the European Convention ETS/STE No. 123 and international standard GLP (OECD Guide 1:1998). Planned euthanasia of animals was performed on an automated CO_2_-euthanizer AVTech (INPREN Ltd., Moscow, Russia).

### 4.2. Drugs

In the study, we compared the properties and effects of two salts of 1-isobutanoyl-2-isopropylisothiourea—the salt formed by hydrobromic acid (compound T1023) and the salt formed by phosphoric acid (compound T1082). Compounds T1023 and T1082 were synthesized in the laboratory of radiation pharmacology of A.F. Tsyb MRRC. T1023 was synthesized by the previously described method [18].

The method for T1082 preparation consisted of the isolation of the free base, 1-isobutanoyl-2-isopropylisothiourea, from compound T1023, which was then reacted with phosphoric acid [37]. The T1082 structure has been confirmed by NMR-spectrometry and elemental analysis. Spectra 1H NMR was obtained in DMSO-d6 on the spectrometer Avance DRX-500 (Bruker BioSpin GmbH, Rheinstetten, Germany) at a frequency of 500 MHz with tetramethylsilane as the internal standard. Elemental analyses of C, H, and N were performed on an elemental analyzer EA 1108 (CE Instruments, Egelsbach, Germany). The control of T1082 purity was performed using thin layer chromatography and melting temperature measurements. The melting point of T1082 was determined on an automatic heating unit PTP-M (LOIP Ltd., St. Petersburg, Russia). Chromatography on Silufol UV-254 plates (Kavalierglass, Sazava, Czech Republic) was performed in a benzene–ethanol–triethylamine (9:1:0.1) system.

T1082 synthesis example: 2.8 g of 1-isobutanoyl-2-isopropylisothiourea hydrobromide was dissolved in 10 mL of water, with a concentrated aqueous solution of ammonia being added dropwise to pH 8 and extracted twice with diethyl ether (2 × 15 mL). Next, the sample was dried with anhydrous Na_2_SO_4_ and then evaporated. The base yield was 1.7 g. The resulting base was dissolved in 10 mL of acetone; next, 0.9 g of phosphoric acid was added dropwise. The sample was then allowed to cool down. The precipitate is filtered off and recrystallized from a large volume of acetone. The yield was 2.2 g (84% in terms of T1023) of a white, crystalline product. Spectra 1H NMR (500 MHz; DMSO-d6, δ): 1.05 (d, 6H); 1.3 (d, 6H); 2.45 (m, 1H); 4.4 (b, 6H). Calculated (%): C 33.55; H 6.68; N 9.78. Found (%): C 33.43; H 6.65; N 9.76. The melting temperature is 129–131 °C (with decomposition). Rf value is 0.35. Molecular weight: 286.4 g/mol. Chemical formula: C_8_H_16_N_2_OS⋅H_3_PO_4_.

The methods used for the synthesis, isolation and purification T1023 and T1084 ensured the stable quality of substances with an active substance content of more than 95% and a total content of related and extraneous impurities of less than 1% of dry weight. Compounds T1023 and T1082 are easily soluble in water, acetone, and chloroform, and insoluble in hexane. One percent aqueous solutions of T1023 and T1082 are transparent and colorless at pH 3.72 and 3.95, respectively. In experiments, T1023 and T1082 were administered to experimental animals by a intraperitoneal (i.p.) or intragastric (i.g.) method once in the form of aqueous solutions, which were prepared *ex tempore* in water for injection (JSC Dalkhimfarm, Khabarovsk, Russia). All control, untreated animals at the same time and in the same way were injected with 0.9% sodium chloride for injection (Dalchimpharm) in an equivalent volume. For i.g. administration, steel bent gastric tubes (Vivariy, Belgorod, Russia) were used: 20 G × 38 mm with olive 2.25 mm for mice; 16 G × 76 mm with olive 3 mm for rats.

### 4.3. Toxicology

T1082 toxicological studies were carried out on outbred mice according to the acute toxicity test with a single i.p. and i.g. administration. For each route of administration, the LD_50_ value was preliminarily estimated by the Deichmann–Leblanc method on a limited number of animals (5 groups of 2 mice). A detailed assessment of the toxicity parameters was carried out by the probit analysis method according to Litchfield and Wilcoxon [38]. For each route of administration, forty white, outbred, male mice were divided into five groups, with eight animals in each group. T1082 was administered i.p. at doses of 300–470 mg/kg (0.2 mL of 1.5–2.4% solution per 10 g of body weight), and i.g. at doses of 2300–3250 mg/kg (0.2 mL of 11.5–16.2% solution per 10 g of body weight). Each animal was monitored individually for 15 days, with special attention given during the first 4 h. All dead and surviving mice (at the end of the observation) underwent a macroscopic examination of the internal organs.

### 4.4. Radiation Exposure

In this study, γ-ray total-body irradiation (TBI) was performed on male F1 (CBA×C57BL6j) mice and β-ray local irradiation (LI) of the right thigh of male Wistar rats. γ-ray TBI of mice at doses of 5 Gy (when studying the survival splenic colony-forming cells) and 9–9.5 Gy (when studying the 30-day survival of mice) were performed at research facility Luch-1 (RPE Gamma, Moscow, Russia) with a ^60^Co source. Its technical specifications and used dosimetric support are described in [18]. For this study, 10–12 mice were placed in an acrylate container with individual cells and irradiated with static position of the γ-ray source (irradiation field 450 × 450 mm, focal length 750 mm) in the dorsal-ventral irradiation geometry at a dose rate of 2.8 Gy/min. According to dosimetry data, the uneven distribution of the γ-ray absorbed dose was in the range of 2–4%.

β-ray LI of rats were performed on a medical electron accelerator Novac-11 (Sordina IORT Technologies, Vicenza, Italy). β-ray dosimetry was performed using a PTW MP3-P water phantom, a PTW Tandem XDR 2-channel dosimeter, and a set of PTW Farmer Chamber Type 30,013 and PTW Roos Chamber Type 3400 ionizing chambers. Dosimetry calculations were carried out using the PTW MEPHYSTO software according to TRS recommendations No. 398. The day before irradiation, the coat was removed on the rat’s right hind limb using a trimmer Moser Chromini Type 1591 (Wahl GmbH, Schwarzwald, Germany). The animal was placed in an individual acrylate container, from which the right hind limb was removed and fixed. The tube of the β-source (diameter 40 mm) was lowered close orthogonally to the lateral surface of the thigh. Irradiation was performed by electrons with energy of 10 MeV single at a dose of 40 Gy and fractionally at a total dose 78 Gy (6 fractions at 13 Gy with a 24-h interval) in the lateral-medial irradiation geometry at dose rate 39 Gy/min. According to dosimetry data, the uneven distribution of the β-ray absorbed dose in tissues of rat’s thigh was in the range of 5–7%.

### 4.5. Schemes of Radiobiological Experiments in Mice and the Evaluation of Their Effects

A comparative study of the radioprotective activity of compounds T1023 and T1082 was carried out in three series of radiobiological experiments with γ-ray TBI of male F1 (CBA×C57BL6j) mice.

In the first series of experiments, using Till–McCulloch’s method [39], the effect of compounds T1023 and T1082 at parenteral (i.p.) administration at equimolar doses was studied, which corresponds to 1/18, 1/12, 1/8, 1/4, and 1/3 LD_10_, on the number of endogenous splenic colony-forming units (CFU) in mice on the 8^th^ day after γ-ray TBI at a dose of 5 Gy. Two independent experiments were performed according to the same scheme, in which 11 groups of mice (*n* = 16–17 in each) were used—the irradiation control group and 10 experimental groups. Mice of the experimental groups were treated preventively (i.p.; 30 min before TBI) with T1023 and T1082 at doses of 63, 93, 149, 278, and 480 µmol/kg (17/18, 25/27, 40/43, 75/80, and 130/138 mg/kg, respectively—0.1 mL of 0.17–1.38% solution per 10 g of body weight), and control mice were given 0.9% sodium chloride in an equivalent volume. Eight days after TBI, euthanasia was performed, the spleens were removed and fixed for 24 h in Bouin’s fluid (BioVitrum Ltd., St. Petersburg, Russia), and the number of endogenous CFU was counted on their surface. For a generalized analysis of these experiments, the data of each experiment were normalized to the mean of CFU in control mice (converted to relative units, in which the number of CFU in the control of each experiment is 1 ± SD). The severity and differences in the radioprotective activity of T1023 and T1082 in these experiments were evaluated by intergroup statistical comparison of the number of CFU in the generalized data.

In the second series of experiments, the effect of compounds T1023 and T1082 at parenteral (i.p.) administration at equimolar doses was studied, which correspond to 1/12, 1/8, and 1/4 LD_10_, on the 30-day survival of mice after γ-ray TBI at a dose of 9.5 Gy. Mice of the experimental groups (*n* = 15–31) were treated preventively (i.p.; 30 min before TBI) with T1023 and T1082 at doses of 93, 149, and 278 µmol/kg (25.0/26.5, 40.0/42.5, and 75.0/79.7 mg/kg, respectively—0.1 mL of 0.25–0.8% solution per 10 g of body weight), and control mice (*n* = 30) were given 0.9% sodium chloride in an equivalent volume. Within 30 days after TBI, the condition of the animals was assessed at a two-fold daily examination and the time of their death was recorded. Further, using Kaplan–Meier’s method, data were plotted to create diagrams of the survival of mice in different groups. The severity and differences in the radioprotective activity of T1023 and T1082 in these experiments were evaluated by an intergroup statistical comparison of the survival diagrams.

In the third series of experiments, a comparative study of the effect of compounds T1023 and T1082 at intragastric (i.g.) administration on the 30-day survival of mice after γ-ray TBI at a dose of 9 Gy was carried out. Mice of the experimental groups (*n* = 20–34) were treated preventively (i.g.; 30 min before TBI) with T1082 at doses 50, 75, 100, 125, and 150 mg/kg (0.1 mL of 0.5–1.5% solution per 10 g of body weight) or with T1023 at doses 125 and 150 mg/kg (0.1 mL of 1.25–1.5% solution per 10 g of body weight), and control mice (*n* = 34) were given 0.9% sodium chloride in an equivalent volume. Registration of data and analysis of the results in these experiments was carried out in the same way as in the case of parenteral administration (in the second series of experiments). Calculation of the “dose-effect” dependence for the radioprotective efficacy of T1082 at i.g. administration and evaluation of ED indicators were carried out using probit regression analysis.

### 4.6. Radiation-Induced Skin Reactions in Rats

The ability of compound T1082 to prevent radiation damage of normal tissues was studied in models of acute RSR on the right thigh of Wistar rats after single β-ray LI at a dose of 40 Gy and fractionated β-ray LI at a total dose of 78 Gy (6 fractions at a dose of 13 Gy with a 24-h interval). Five groups of animals were used, with three rats in each group. Animals of groups 1–2 received single LI. Rats of the 1st group were preventively treated with T1082 (30 min before LI; i.g., 160 mg/kg—1 mL of 0.8% solution per 100 g of body weight), and rats of the 2nd group were injected (i.g.) with 2 mL of 0.9% sodium chloride. Animals of groups 3–4 received fractionated LI. In addition, before each irradiation fraction, these rats were treated with T1082 and sodium chloride at the same time and in the same doses as the rats of groups 1–2. Rats of the 5th group (non-irradiated control) were injected (i.g.) with 2 mL of 0.9% sodium chloride.

After the beginning of the experimental exposure, the clinical and morphological manifestations of acute radiation damage to the skin and its appendages were studied in all irradiated, untreated, and treated T1082 rats during daily examinations. The nature of the course of inflammatory and regenerative processes and the severity of RSR were assessed according to the six-point clinical scale RTOG/EORTC-1995 [26,27]. Euthanasia was performed at the time when the clinical and morphological manifestations of RSR reached the maximum severity in irradiated, untreated rats (on the 11th day after a single LI and on the 15th day after the beginning of fractionated LI). After euthanasia, biological materials for pathomorphological studies were taken. From each animal, two transverse skin fragments with underlying tissues about 2 cm long were isolated at the border of the most damaged and visually unchanged skin from the lateral and medial surfaces of the thigh, as well as a part of the greater curvature of the stomach and jejunum.

The isolated materials were fixed for 24 h in Bouin’s liquid (BioVitrum Ltd.) and washed with 70% ethanol. After standard histological processing on a Leica TP1020 carousel histoprocessor (Leica Biosystems Inc., Buffalo Grove, IL, USA), tissue samples oriented along the long axis were dehydrated and embedded in the Histomix paraffin medium (Sakura Finetek USA, CA, USA) at the HistoStar filling station (Thermo Fisher Scientific, Waltham, MA, USA). For morphological studies, sections with a thickness of 5 μm, obtained with a Leica RM2235 microtome (Leica Biosystems Inc.), were stained with hematoxylin and eosin (H&E; Biovitrum Ltd., St. Petersburg, Russia) after deparaffinization. Histological sections of tissues of the control, irradiated T1082-untreated and irradiated T1082-treated rats, stained with H&E, were examined under an Axio Imager A1 microscope (Zeiss AG, Jena, Germany) at three objective magnification levels (×5, ×10, and ×40) with microphotography of images being taken on a Power Shot A640 digital camera (Canon Inc., Tokyo, Japan). The results of the studies obtained in the irradiated T1082-untreated and T1082-treated groups were compared with the data obtained in rats of the non-irradiated control group. The pathomorphological analysis was performed, taking into account the criteria for variants of the norm and pathology, as well as artificial, supravital, and reactive changes in cells caused by manipulations during tissue isolation and their fixation [40,41].

### 4.7. Statistical Analysis

Standard parameters of variation statistics were calculated for all experimental data and their values are given as M ± SD. The significance of intergroup differences in the number of CFUs was assessed in multiple comparisons using the Kruskal–Wallis ANOVA by ranks with a post hoc Mann–Whitney U test by Bonferroni–Holm multiple test procedure [42], and in pair comparisons using the Mann–Whitney U test. The significance of intergroup differences in survival diagrams was assessed in multiple comparisons using χ^2^-test with post hoc Cox F test by a Bonferroni–Holm multiple test procedure, and in pair comparisons using the Cox F test. Statistical significance of regressions was assessed using χ^2^ criterion. In all cases, the effects and differences were considered statistically significant at a 5% level. The statistical calculations were performed using the software packages Statistica 10 (StatSoft Inc., OK, USA) and BioStat 7.3 (AnalystSoft Inc., Alexandria, CA, USA).

## 5. Conclusions

The results confirm the significant interest of NOS inhibitors for radiation medicine and oncology, and allow T1082 to be considered as one of the safest radioprotectors with emergency preventive action, as well as a possible pharmacological agent for the prevention of RT complications.

## Figures and Tables

**Table 1:** Indicators of T1082 and T1023 acute toxicity for outbred mice after a single intraperitoneal and intragastric administration.

Compound, Route of Administration	LD_10/15_		LD_16/15_		LD_50/15_		LD_84/15_	
	mg/kg	mM	mg/kg	mM	mg/kg	mM	mg/kg	mM
T1023, i.p. *	317	1.2	333	1.2	410	1.5	488	1.8
T1082, i.p.	321	1.1	338	1.2	403	1.4	481	1.7
T1082, i.g.	2290	8.0	2364	8.3	2638	9.2	2944	10.3

* indicators for compound T1023 obtained earlier [17,18].

**Table 2:** Pharmacological safety indicators of T1082 radioprotective action in mice after intragastric administration.

Compound, Route of Administration	LD_10_/ED_16_	LD_50_/ED_50_	LD_10_/ED_50_	LD_10_/ED_opt_
T1082, i.g.	42.1	30.1	26.1	10.2–16.2

## Data Availability

Not applicable.
